# Functional Validation of *ALDOA* in Regulating Muscle Cell Fate: Based on In Vitro Proliferation, Apoptosis, and Differentiation Experiments

**DOI:** 10.3390/genes16101186

**Published:** 2025-10-12

**Authors:** Hongzhen Cao, Jing Wang, Yunzhou Wang, Jingsen Huang, Wei Chen, Hui Tang, Junfeng Chen, Baosong Xing, Yongqing Zeng

**Affiliations:** 1 Shandong Provincial Key Laboratory for Livestock Germplasm Innovation & Utilization, College of Animal Science and Technology, Shandong Agricultural University, Tai’an 271018, China; hongzhencao@163.com (H.C.); hjs990606@163.com (J.H.); wchen@sdau.edu.cn (W.C.); tanghui@sdau.edu.cn (H.T.); 2 Henan Key Laboratory of Farm Animal Breeding and Nutritional Regulation, Henan Pig Breeding Engineering Research Centre, Institute of Animal Husbandry, Henan Academy of Agricultural Sciences, Zhengzhou 450002, China; wangjing@hnagri.org.cn (J.W.); afeng008@163.com (J.C.); 3 Department of Veterinary Medicine, Shandong Vocational Animal Science and Veterinary College, Weifang 261061, China; wyzwin01@163.com

**Keywords:** C2C12 cells, skeletal muscle satellite cells, *ALDOA* gene

## Abstract

**Background/Objectives**: This study systematically investigated the expression characteristics of the *ALDOA* gene in skeletal muscle cells and its effects on cell proliferation, apoptosis, and differentiation. **Methods**: We constructed an *ALDOA* overexpression vector and transfected it into C2C12 cells and porcine skeletal muscle satellite cells. **Results**: We found that *ALDOA* exhibited the highest expression in the longissimus dorsi muscle and was primarily localized in the cell nucleus. Overexpression of *ALDOA* significantly inhibited cell proliferation, induced G0/G1 phase arrest, and downregulated the expression of proliferation-related genes such as *CDK2* and *Cyclin D1*. Concurrently, *ALDOA* overexpression markedly promoted apoptosis. Regarding differentiation, although *ALDOA* expression was upregulated during differentiation, its overexpression significantly suppressed the expression of myogenic differentiation-related genes (such as *MYOD*, *MYOG*, *MEF2C*), suggesting a negative regulatory role in differentiation control. **Conclusions**: This study reveals the multifaceted regulatory functions of *ALDOA* in skeletal muscle cells, providing experimental evidence for deepening the understanding of its mechanisms in muscle development and regeneration. This study provides the first functional evidence that *ALDOA* acts as a multifunctional regulator in skeletal muscle cells, negatively governing cell growth and fate decisions by inhibiting proliferation, promoting apoptosis, and impeding myogenic differentiation, thereby extending its role beyond glycolysis to direct governance of cellular processes. This study reveals for the first time that *ALDOA* possesses dual functions in muscle cells, regulating both metabolism and transcription.

## 1. Introduction

Aldolase A (*ALDOA*) is a key enzyme in the glycolytic pathway, catalyzing the cleavage of 1,6-diphosphate fructose into 3-phosphoglycerate and dihydroxypropyl phosphate [1]. The *ALDOA* gene is located at the human 16p11.2 locus. The protein it encodes is highly expressed primarily in tissues with high energy demands, such as skeletal muscle, cardiac muscle, and red blood cells. Its expression levels are precisely regulated during processes including embryonic development, tissue regeneration, and tumorigenesis [2]. Traditional views hold that *ALDOA* is solely involved in energy supply. However, mounting evidence indicates that *ALDOA*’s functions extend far beyond its classical role as a glycolytic enzyme. As a multifunctional protein, it extensively participates in regulating processes such as cytoskeletal dynamics, stress responses, and gene transcription—particularly in cell types with high energy metabolism, such as skeletal muscle cells [3]. *ALDOA* also regulates cell cycle progression, redox balance, cytoskeletal dynamics, and gene transcription through “non-classical” mechanisms, thereby profoundly influencing cell proliferation, apoptosis, and differentiation [4]. In recent years, the “moonlighting functions” of metabolic enzymes within the cell nucleus have garnered increasing attention. Accumulating evidence indicates that multiple metabolic enzymes, including hexokinase, pyruvate kinase M2, and aldolase A, can localize to the nucleus. Through mechanisms such as regulating transcription factor activity, chromatin modification, and DNA damage repair, these enzymes directly participate in gene expression regulation [5]. This provides important theoretical background for the potentially similar direct nuclear regulatory role of *ALDOA* in muscle cells.

Research indicates that skeletal muscle serves as the primary site for glucose storage and insulin uptake, playing a crucial role in systemic metabolism [6]. The development, regeneration, and homeostasis of skeletal muscle represent a finely coordinated process involving the activation, proliferation, differentiation, and apoptosis of muscle satellite cells. Myogenesis commences with the proliferation of myoblasts, followed by their exit from the cell cycle to initiate differentiation programs, culminating in the fusion of these cells to form multinucleated myotubes [4,7,8]. This series of biological events requires substantial energy and metabolic reprogramming, with the dynamic changes in glycolytic activity serving as a key regulatory switch for myogenic processes [9,10]. During the proliferation phase, myoblasts rely on high glycolytic activity to rapidly generate ATP and biosynthetic precursors. In contrast, the metabolic profile shifts during differentiation, yet a sustained glycolytic flux remains crucial for myotube maturation [11]. Therefore, as a core enzyme in glycolysis, *ALDOA* may play a central regulatory role in this metabolic network that intersects with cell fate determination.

Previous studies have revealed the “non-classical” functions of *ALDOA* in multiple cancer models. For instance, *ALDOA* promotes tumor cell migration and invasion by binding to actin (F-actin) and playing a role in cell membrane ruffling and motility [12]. Although it has been demonstrated that *ALDOA* regulates the cell cycle and migration in various types of cancer, its specific functions in muscle biology, particularly in the determination of the fate of satellite cells, remain unclear. Resolving this question is crucial for deepening our understanding of muscle physiology and the pathological mechanisms underlying related diseases, such as rhabdomyolysis, myopathies, and muscular atrophy [13].

Against this background, this study aims to systematically investigate the direct effects of the *ALDOA* gene on myoblast proliferation, apoptosis, and differentiation at the cellular level. By overexpressing *ALDOA* in mouse C2C12 myoblasts and porcine skeletal muscle satellite cells, we comprehensively evaluated the phenotypic effects of *ALDOA* overexpression on myogenic processes using key techniques including CCK-8 proliferation assays, flow cytometry analysis of cell cycle and apoptosis, and immunofluorescence staining. This study provides novel insights into the function of *ALDOA* in muscle biology, elevating its role beyond that of a traditional metabolic enzyme to a multifunctional node integrating metabolic signaling with cellular behavioral decisions. It also offers a potential theoretical basis for targeting metabolism to treat muscle-related diseases.

## 2. Materials and Methods

### 2.1. Cell Culture and Differentiation

The experimental pigs were selected from *Jiangquan black pigs* from a breeding farm in Shandong Province, and two *JBPs* with high and low average daily gain were screened according to average daily gain for single-cell nuclear sequencing. The genetic screening was based on the results of single-cell nuclear sequencing in our laboratory, and the mouse muscle satellite cell line C2C12 (purchased from Wuhan Prosperity Life Sciences Co., Ltd.) and pig primary skeletal muscle cells were used for the experiment. The medium of C2C12 muscle satellite cells was supplemented with 10% fetal bovine serum (FBS), 1% antibody mixture of penicillin, streptomycin, and gentamycin, and high-sugar Dulcimer’s modified medium (DMEM, Gibco). Primary porcine skeletal muscle cells were modified to a medium containing 20% FBS, supplemented with 0.5% chicken embryo extract (CEE) (Solarbio, S9080, Beijing, China), 1% non-essential amino acid solution (NEAA) (Gibco, 11140050, Grand Island, NY, USA), 1% L-glutamine (Solarbio, IG0390, Beijing, China), and 2.5 µL of basic fibroblast growth factor (bFGF) (Solarbio, CLP0338, Beijing, China) per 100 mL. The culture was incubated at 37 °C in an incubator containing 5% CO_2_ at the same time. When cells grew to 60–80%, differentiation medium (2% horse serum (HS)) was added to induce them to undergo differentiation, and samples were taken at different periods, with the start of induction recorded as D0, and cells differentiating D1, D3, D5, D7, and D9 were collected for subsequent experiments.

### 2.2. Analysis of ALDOA Sequence Information

The cDNA was synthesized by designing primers based on the sequence of the *ALDOA* gene in NCBI and the primer sequences (see Table 1), and the full length of the gene was amplified with ApexHF HS DNA polymerase CL (AG12204), after which the PCR products were gel recovered (TIANGEN, DP209, Beijing, China) and sequenced, and the results obtained were subjected to blast comparison and then analyzed for conserved regions as well as evolutionary relationships by MEGA-X to analyze the conserved regions and evolutionary relationships of the genes.

### 2.3. Cellular Immunofluorescence

Cells were inoculated onto 6-well plate cell crawlers at a density of 1 × 10^5^ cells/mL, cultured until the desired number of days, removed, and rinsed three times in pre-cooled PBS at 4 °C; cells were then fixed with pre-cooled 4% paraformaldehyde fixative for 30 min at 37 °C and rinsed three times in PBS; cells were permeabilized with 0.1% Triton X-100 for 40 min at room temperature, and rinsed three times in PBS. After removing the PBS, the cells were closed with 5% fetal bovine serum at 37 °C for 1 h; after removing the closure solution, the primary antibody (1:1000 dilution) was added and incubated at 4 °C overnight; the cells were rinsed three times with PBS, then the Alexa fluor 647 secondary antibody (1:1000 dilution) was added, and they were incubated for 1 h at 37 °C, avoiding light; the cells were rinsed with PBS three times; after removing the PBS, the cells were stained with 200 µL of DIPA staining solution dropwise, and incubated for 10 min at 37 °C, avoiding light. After removing the PBS, 200 µL of DIPA staining solution was added dropwise and incubated at 37 °C for 10 min, and the cells were rinsed with PBS buffer 3 times. Finally, the cell crawls were placed upside down on ordinary slides. The images were viewed, captured and saved with a laser confocal microscope.

### 2.4. Plasmid Construction and Cell Transfection

The full-length cDNA of *ALDOA* was obtained via PCR. The PCR product underwent gel electrophoresis and gel-based sequencing, followed by sequence alignment with NCBI database entries to verify sequence accuracy. After digestion of PCR products and plasmids, the *ALDOA* fragment was ligated into the PCDNA3.1 vector using T4 DNA ligase, generating ALDOA-PCDNA3.1. Sequence accuracy was verified by sequencing. Ten microliters of recombinant plasmid were added to 50 µL of DH5α receptor cells. Following transformation, culture, and amplification, plasmid was extracted for subsequent experiments. For transfection, the expression vector was transfected into C2C12 cells and porcine skeletal muscle primary cells using a lipid transfection reagent (Next Sage Biologics). Cells were seeded in 6-well plates prior to transfection. When cell confluence reached 60–80%, 5 µg of the overexpression vector was transfected into the cells. Transfection efficiency was assessed using empty vector transfection, with all groups maintaining transfection efficiency above 70%.

### 2.5. Cell Proliferation Assay

The proliferative activity of the cells was calculated using the cck-8 kit, the cell suspension was added to 96-well plates at 100 µL/well, 6 replicates were performed for each group, and an equal amount of complete medium was added to the 6 blank wells as a blank control. After 12 h of cell culture, the cells were transfected with interference fragments and overexpression vectors. The culture was continued, and the cell viability at 12 h, 24 h, 36 h and 48 h is determined with the end of transfection as the time node. In the assay, 10 µL of cck-8 solution was added to each well, the cells were cultured in an incubator protected from light for 2 h, and the absorbance at 450 nm was measured by an enzyme marker.

To observe the cell proliferation viability using the Edu-488 kit, cell crawls were placed in 6-well plates, followed by the addition of 200 µL of cell suspension dropwise to each crawl, and after 1 h of incubation, 1.5 mL of growth medium was slowly added to each well, and the cells were transfected when the cell density reached 70–80%. After 24 h of dropwise addition of Edu-488 reagent and 2 h of incubation, the subsequent operations were carried out, and all subsequent operations were carried out according to the instructions of the test kit. 

### 2.6. Detection of Cell Proliferation and Apoptosis by Flow Cytometry

Cells were spread on the plate and transfected, and after the desired amount of cells was reached, experiments were carried out with the Cell Cycle and Apoptosis Detection Kit (Beyotime, C1052, Shanghai, China) and the Annexin V-FITC Apoptosis Detection Kit (Beyotime, C1062M, Shanghai, China), and all the operations were carried out according to the instructions. After collecting the desired cells, the results were analyzed using FlowJo v10.8.1 software to analyze the results. All experiments were performed in triplicate.

### 2.7. Real-Time Quantitative Fluorescence Detection

RNA Extraction Kit (TIANGEN, RNA simple Total RNA Extraction Kit), Reverse Transcription Kit (Evo M-MLV Reverse Transcription Premix Kit), and SYBR^®^ Green Pro Taq HS Pre-mixed qPCR Kit were obtained from Accurate Biotechnology, and the entire experiment was performed on ice. Experiments were performed by adding 1 µg of total RNA to 20 µL of reaction mixture, synthesizing first-strand cDNA using reverse transcriptase, and reversing using a two-step method, with the first step having a reaction time of 2 min at 42 °C, the second step having the following reaction times: 15 min at 37 °C; 5 s at 85 °C, and storage at 4 °C. The first step was performed using a two-step method. 2 µM (0.4 µL) and SYBR Premix Ex Taq (2×) (10 µL) were used for gene expression analysis. All reactions were performed in triplicate and the relative amount of gene expression was calculated and normalized to the control using 2^−ΔΔCt^, where Δ Ct = Ct gene − Ct control. β-actin was used as an internal control. The primers used are detailed in Table 2. All experiments were performed in triplicate.

### 2.8. Western Blot

After raising the treated cells to the desired number of days, they were lysed using RIPA lysis solution containing PMSF (Beyotime, Cat No. P0013B, Shanghai, China). It was collected into a 1.5 mL centrifuge tube, mixed thoroughly and centrifuged at 4 °C, 12,000 rpm for 5 min to collect the supernatant. The concentration of the proteins was assayed by BCA protein assay kit (Beyotime, Shanghai, China). The protein samples were diluted to the same concentration with RIPA, and 5× SDS-PAGE up sampling buffer was added proportionally, then heated with a metal bath at 100 °C for 10 min, and stored temporarily in a refrigerator at −20 °C for backup. Protein electrophoresis was carried out using elife pre-made gel at the required voltage; the protein was transferred to the membrane (to avoid air bubbles) and closed (37 °C, 30 min) using NCM Biotech Rapid Membrane Transfer Liquid, followed by the addition of primary antibody (1:1000 dilution), and overnight incubation at 4 °C. After recovering the primary antibody, it was washed with 1× TBST, followed by the addition of secondary antibody (1:1000 dilution) and incubated at room temperature for 2 h. The protein bands were detected by chemiluminescence imaging with ECL luminescent solution (Beyotime) after washing with TBST.

### 2.9. Statistical Analysis

Protein grayscale was determined using Image J v1.54 software, all data were analyzed using IBM SPSS Statistics 25 software, and the statistical significance of differences between groups was determined using the independent samples *t*-test or one-way ANOVA test. The images were also plotted using GraphPad Prism 8.0, and the data of each group were expressed as mean ± SEM. Statistical significance was expressed as * *p* < 0.05; ** *p* < 0.01; *** *p* < 0.001.

## 3. Result

### 3.1. Analysis of ALDOA Gene Sequence Information

**Figure 1:**
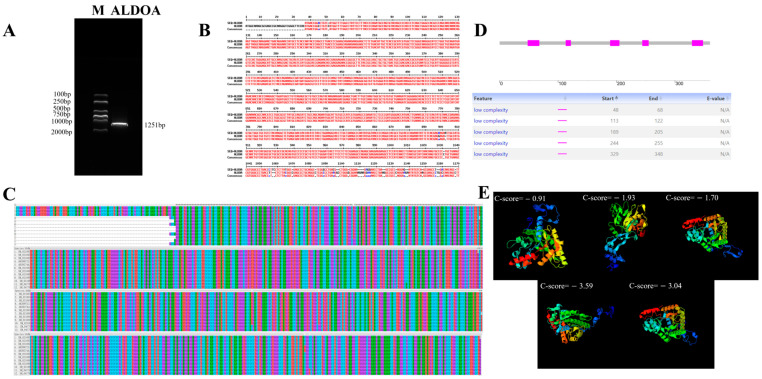
Analysis of *ALDOA* Gene Sequence Information. (**A**): PCR amplification results of the coding region; (**B**): Comparison of amplified sequencing results with NCBI entries; (**C**): Conserved sequence alignment of the *ALDOA* gene; The “*” in (**C**) indicates the conserved sequence of this gene. (**D**): Smart prediction of *ALDOA* functional domains; (**E**): Predicted tertiary structure of the *ALDOA* gene. Estimated TM-score = 0.60 ± 0.14; Estimated RMSD = 8.6 ± 4.5 Å. Analysis of Basic Traits of the *ALDOA* Gene.

In pigs, the cDNA length of the *ALDOA* gene is 2359 bp, encompassing a 1251 bp open reading frame (ORF) (Figure 1A). Sequence alignment between the amplified ORF region and NCBI entries demonstrated high amplification fidelity (Figure 1B) (Supplementary Materials Figure S1). Subsequently, we randomly downloaded 12 genes from NCBI for sequence comparison and found that the *ALDOA* gene exhibits high conservation across different species, providing a theoretical basis for selecting diverse cell types (Figure 1C) (Supplementary Materials Figure S2). Smart domain prediction identified five conserved functional domains within the ORF region (Figure 1D). It encodes a protein composed of 360 amino acid residues, with a molecular weight of 39,267.37 daltons and an isoelectric point of 11.50. Within the encoded protein, the number of coil structures significantly exceeds that of chain structures, while the number of helix structures is intermediate. Based on the protein’s secondary structure, five *ALDOA* tertiary structures were predicted (Figure 1E).

**Figure 2:**
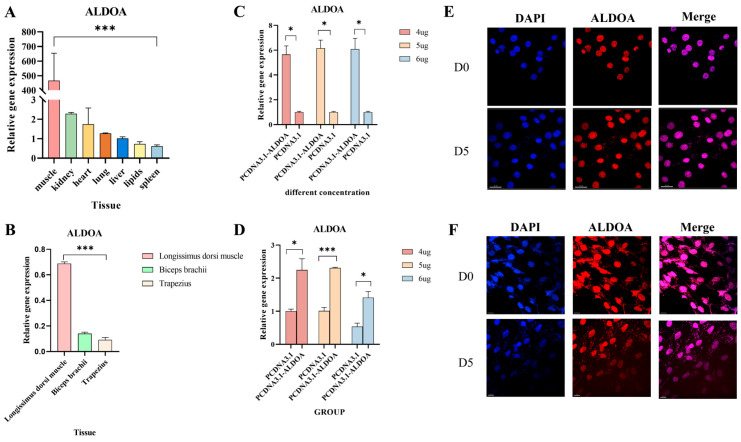
Analysis of Gene Basic Traits. (**A**): Expression levels of the *ALDOA* gene in different tissues; (**B**): Expression levels of the *ALDOA* gene in different muscle tissues; (**C**): Expression levels of the *ALDOA* gene at different concentrations in C2C12 cells; (**D**): Expression levels of the *ALDOA* gene at different concentrations in porcine skeletal muscle satellite cells; (**E**): Localization of the *ALDOA* gene in C2C12 cells at different time points; (**F**): Localization of the *ALDOA* gene in porcine skeletal muscle satellite cells at different time points (* *p* < 0.05, *** *p* < 0.001).

During sampling, visceral organs such as the heart, liver, and kidneys were preserved. After extracting RNA from these tissues for expression profiling, it was found that the *ALDOA* gene exhibited the highest expression levels in muscle tissue, significantly exceeding those in other visceral tissues and adipose tissue (*p* < 0.001) (Figure 2A). Simultaneously, quantitative analysis of muscle tissues from different regions revealed that *ALDOA* gene expression was highest in the longissimus dorsi muscle, significantly exceeding levels in the biceps brachii and trapezius muscles (*p* < 0.001) (Figure 2B). After constructing an overexpression vector, preliminary experiments on vector loading concentrations revealed that the selected concentration significantly increased expression levels in C2C12 cells compared to the empty vector control (*p* < 0.05) (Figure 2C). In porcine skeletal muscle satellite cells, the most pronounced effect was observed at a vector concentration of 5 µg, with the overexpression group showing a highly significant increase compared to the empty vector group (*p* < 0.001) (Figure 2D). Subsequently, C2C12 and porcine skeletal muscle satellite cells were transfected and subjected to immunofluorescence staining. Results revealed that the *ALDOA* gene was expressed in the cell nucleus at both D0 and differentiation day 5 (D5) (Figure 2E,F) (Supplementary Materials Figures S3 and S4).

### 3.2. Effects of ALDOA Overexpression on Skeletal Muscle Cell Proliferation

#### 3.2.1. Effects of *ALDOA* Overexpression on C2C12 Cell Proliferation

**Figure 3:**
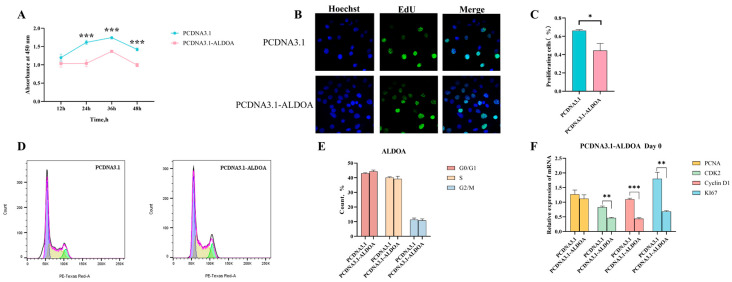
Effects of *ALDOA* Gene Overexpression on C2C12 Cell Proliferation. (**A**): CCK-8 assay for post-transfection cell viability; (**B**): EdU assay for cell proliferation; (**C**): Proportion of proliferating cells calculated from EdU fluorescence results; (**D**): Flow cytometry analysis of cell cycle distribution; (**E**): Proportion of cells in each cell cycle phase; (**F**): Quantitative results of proliferation markers (* *p* < 0.05, ** *p* < 0.01, *** *p* < 0.001).

Following transfection of cells with the overexpression vector, CCK-8 assays were performed at 12 h, 24 h, 36 h, and 48 h post-transfection. Results revealed that at 24 h post-transfection, the cell viability of the empty vector control group was significantly higher than that of the experimental group (*p* < 0.001) (Figure 3A). Concurrently, EdU experiments visually demonstrated that the proportion of proliferating cells was significantly reduced in the overexpression group compared to the empty vector group (*p* < 0.05) (Figure 3B,C) (Supplementary Materials Figure S5). To further investigate the mechanism, flow cytometry analysis revealed increased G0/G1 phase cells and decreased S phase cells following overexpression. This suggests that *ALDOA* overexpression may induce G0/G1 phase arrest, thereby reducing cell proliferation (Figure 3D,E). Finally, quantitative analysis of cell proliferation-related marker genes revealed significantly reduced expression of *CDK2*, *Cyclin D1*, and *KI67* following overexpression (*p* < 0.01) (Figure 3F). Collectively, these experiments demonstrate that *ALDOA* overexpression reduces cell proliferation.

#### 3.2.2. Effects of *ALDOA* Overexpression on Proliferation of Porcine Skeletal Muscle Satellite Cells

**Figure 4:**
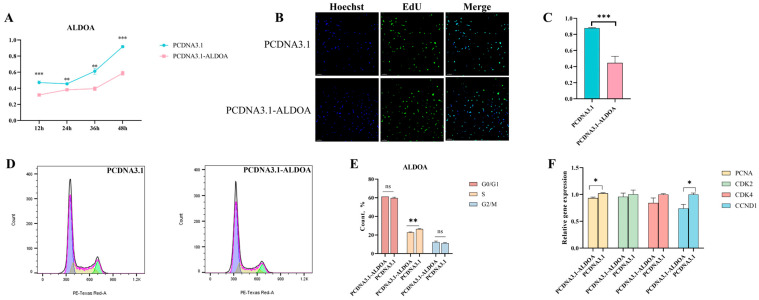
Effects of *ALDOA* Gene Overexpression on Proliferation of Porcine Skeletal Muscle Satellite Cells. (**A**): CCK-8 assay for post-transfection cell viability; (**B**): EdU assay for cell proliferation; (**C**): Proportion of proliferating cells calculated based on EdU fluorescence results; (**D**): Flow cytometry analysis of cell cycle distribution; (**E**): Proportion of cells in each cell cycle phase; (**F**): Quantitative results of proliferation markers (* *p* < 0.05, ** *p* < 0.01, *** *p* < 0.001, “ns” means *p* > 0.05).

For porcine skeletal muscle satellite cells, we performed the same experiments and found that in the CCK-8 assay, the cell viability of the empty vector group at all time points was significantly higher than that of the overexpression group (*p* < 0.01), with a progressive increase in cell absorbance (Figure 4A). This indicates that cells continued to proliferate post-transfection, but their proliferative capacity was diminished. EdU staining revealed a highly significant reduction (*p* < 0.001) in the number of proliferating cells among those transfected with the overexpression vector (Figure 4B,C) (Supplementary Materials Figure S6). Flow cytometric analysis of the cell cycle showed no significant difference in the proportion of cells in the G0/G1 phase. However, the proportion of cells in the S phase was significantly higher in the empty vector group than in the overexpression group (*p* < 0.01) (Figure 4D,E). For cell proliferation-related markers, quantitative analysis revealed that although *CDK2* and *CDK4* expression levels in the overexpression group were lower than those in the control group, the differences did not reach statistical significance (*p* > 0.05). However, *PCNA* and *CCND1* expression was significantly reduced in the overexpression group (*p* < 0.05) (Figure 4F). These findings indicate that *ALDOA* overexpression does not uniformly regulate proliferation-related genes in porcine primary satellite cells. *Cyclin D1* and *PCNA* exhibit heightened sensitivity, whereas the regulation of *CDK2* and *CDK4* may depend on more complex signaling environments or compensatory mechanisms.

### 3.3. Effects of ALDOA Overexpression on Skeletal Muscle Cell Apoptosis

**Figure 5:**
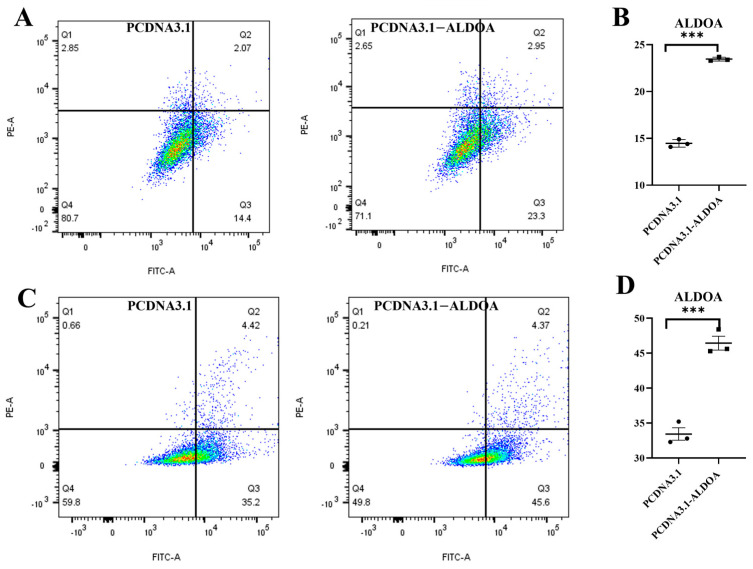
Effects of *ALDOA* Gene Overexpression on Skeletal Muscle Cell Apoptosis. (**A**): Flow cytometry analysis of C2C12 cell apoptosis; (**B**): Analysis of C2C12 cell apoptosis ratio; (**C**): Flow cytometry analysis of skeletal muscle satellite cell apoptosis; (**D**): Analysis of porcine skeletal muscle satellite cell apoptosis ratio (*** *p* < 0.001).

Cells were harvested 48 h after transfection for apoptosis detection. Flow cytometry analysis revealed that following *ALDOA* overexpression, apoptosis rates were significantly higher than in the control group in both C2C12 cells and porcine skeletal muscle satellite cells (*p* < 0.001) (Figure 5A–D). The overexpression level of *ALDOA* is approximately 3 to 4 times that of its endogenous level, which falls within the range of physiological variations. This indicates that overexpression of the *ALDOA* gene may promote cellular apoptosis.

### 3.4. Effects of ALDOA Overexpression on Skeletal Muscle Cell Differentiation

#### 3.4.1. Effects of *ALDOA* Overexpression on *C2C12* Cell Differentiation

**Figure 6:**
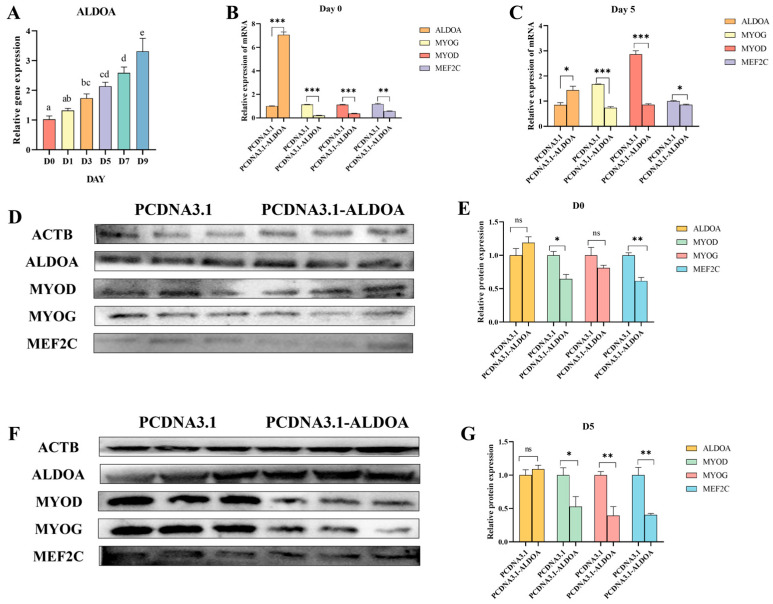
Effects of *ALDOA* Gene Overexpression on C2C12 Cell Differentiation. (**A**): Expression levels of the *ALDOA* gene at different differentiation days; In (**A**), the letters “a b c d e” denote: Identical letters indicate no significant difference (*p* > 0.05)—Different letters indicate significant differences (*p* < 0.05) (**B**): Effects of *ALDOA* gene overexpression at Day 0 on C2C12 cell differentiation; (**C**): Effects of *ALDOA* gene overexpression at Day 5 on C2C12 cell differentiation; (**D**): Western Blot detection of gene expression levels in C2C12 cells at Day 0 of differentiation; (**E**): Protein-level quantification at Day 0; (**F**): Western Blot analysis of gene expression levels in C2C12 cells at Day 5 of differentiation; (**G**): Protein-level quantification at Day 5 (* *p* < 0.05, ** *p* < 0.01, *** *p* < 0.001, “ns” means *p* > 0.05).

When cells reached approximately 80% confluence on 6-well plates, they were induced to differentiate. After collecting cells at different time points and analyzing quantitative results, it was found that the *ALDOA* gene expression showed an increasing trend with the duration of differentiation. Starting from Day 3, a significant difference was observed compared to Day 0 (*p* < 0.05) (Figure 6A). Building on this, we transfected cells with overexpression vectors and empty plasmids, collected cells at D0 and D5, extracted their RNA and proteins for analysis. Results revealed that at D0, *ALDOA* gene expression in the treatment group was significantly higher than in the control group (*p* < 0.01). However, expression levels of differentiation marker genes *MYOG*, *MYOD*, and *MEF2C* were significantly higher in the control group than in the treatment group (*p* < 0.01) (Figure 6B). At D5, gene expression patterns resembled those observed at D0 (Figure 6C). Analysis of protein results revealed that at D0, there was no significant difference in ALDOA and MYOG expression between the treated and control groups. For the differentiation marker gene MYOD, the control group showed significantly higher expression than the treated group (*p* < 0.05). MEF2C expression was significantly higher in the control group than in the treated group (*p* < 0.01) (Figure 6D,E). At D5, ALDOA expression in the treated group was higher than in the control group but not significantly different (*p* > 0.05). MYOD expression in the control group was significantly higher than in the treated group (*p* < 0.05), while both MYOD and MEF2C expression in the control group were extremely significantly higher than in the treated group (*p* < 0.01) (Figure 6F,G). These results indicate that the *ALDOA* gene may inhibit C2C12 cell differentiation.

#### 3.4.2. Effects of *ALDOA* Overexpression on the Differentiation of Porcine Skeletal Muscle Satellite Cells

**Figure 7:**
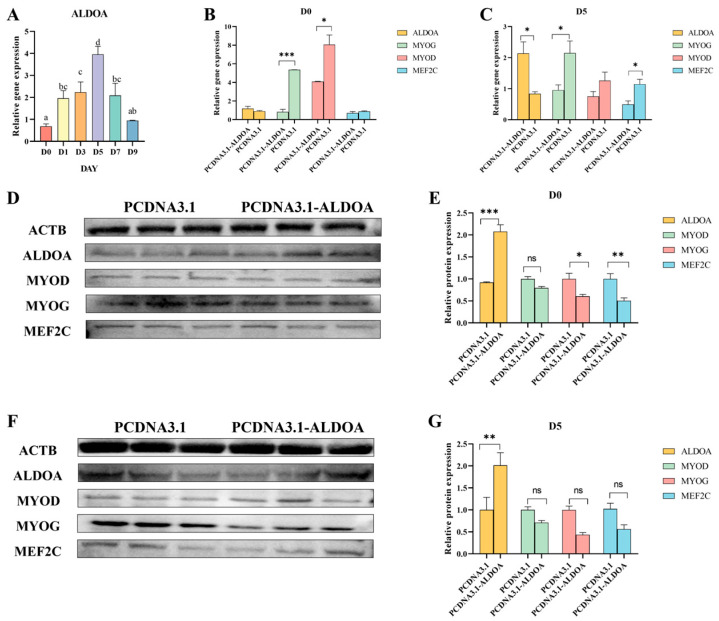
Effects of *ALDOA* Gene Overexpression on Porcine Skeletal Muscle Satellite Cell Differentiation. (**A**): Expression levels of the *ALDOA* gene at different differentiation days; In (**A**), the letters “a b c d” denote: Identical letters indicate no significant difference (*p* > 0.05)—Different letters indicate significant differences (*p* < 0.05) (**B**): Effects of *ALDOA* gene overexpression at Day 0 on porcine skeletal muscle satellite cell differentiation; (**C**): Effects of *ALDOA* gene overexpression at Day 5 on porcine skeletal muscle satellite cell differentiation; (**D**): Western Blot detection of gene expression levels in porcine skeletal muscle satellite cells at Day 0 of differentiation; (**E**): Protein-level quantification at Day 0; (**F**): Western Blot detection of gene expression levels in porcine skeletal muscle satellite cells at Day 5 of differentiation; (**G**): Protein-level quantification at Day 5. (* *p* < 0.05,** *p* < 0.01,*** *p* < 0.001, “ns” means *p* > 0.05).

Quantitative analysis of *ALDOA* gene expression in porcine skeletal muscle satellite cells at different differentiation days revealed peak expression at D5, showing significant difference compared to D0 (*p* < 0.05) (Figure 7A). Quantitative results at differentiation day 0 showed differences between the control and treated groups for *ALDOA* and *MEF2C* genes, though not statistically significant (*p* > 0.05). For *MYOG* expression, the control group was extremely significantly higher than the treated group (*p* < 0.001), while *MYOD* expression was significantly higher in the control group than in the treated group (*p* < 0.05) (Figure 7B). On Day 5 of differentiation, *ALDOA* gene expression was significantly higher in the treatment group than in the control group (*p* < 0.05). However, for differentiation marker genes *MYOG* and *MEF2C*, the control group showed significantly higher expression than the treatment group (*p* < 0.05). There was no significant difference in *MYOG* expression between the control and treatment groups (*p* > 0.05) (Figure 7C). Western blot analysis revealed that at differentiation day 0, ALDOA gene expression was extremely significantly higher in the treatment group than in the control group (*p* < 0.001). MYOD gene expression was higher in the control group than in the treatment group, but the difference was not significant (*p* > 0.05). MYOG expression was significantly higher in the control group than in the treatment group (*p* < 0.05), and MEF2C expression was extremely significantly higher in the control group than in the treatment group (*p* < 0.01) (Figure 7D,E). At day 5 of differentiation, the target gene ALDOA showed significantly higher expression in the treatment group than in the control group (*p* < 0.01). The differentiation marker genes MYOD, MYOG, and MEF2C exhibited higher expression in the control group than in the treatment group, but the difference between the two groups was not significant (*p* > 0.05) (Figure 7F,G).

## 4. Discussion

This study found that the *ALDOA* gene exhibited the highest expression levels in the longissimus dorsi muscle of pigs, significantly exceeding those in other tissues (*p* < 0.05), revealing its important physiological function in skeletal muscle. This result aligns with previous research, indicating that the *ALDOA* gene is highly expressed in tissues with vigorous energy metabolism to meet their high demand for glycolytic products [14]. Furthermore, the *ALDOA* gene is primarily localized to the cell nucleus in C2C12 and porcine skeletal muscle satellite cells, suggesting its potential involvement in nuclear non-metabolic functions such as transcriptional regulation or chromatin remodeling [15]. At the same time, we found that Chen Haidi et al. proposed that *ALDOA* may function in the nucleus, potentially influencing transcription, which aligns with our observation of its nuclear localization [16]. Traditionally regarded as a purely glycolytic enzyme, *ALDOA* has now been identified as a member of a metabolic enzyme family with “secondary functions,” capable of directly participating in gene expression regulation within the cell nucleus. This review summarizes that nuclear *ALDOA*, by binding to AT-rich DNA sequences, participates in processes such as S-phase gene activation, DNA damage protection, and transcript stabilization [5]. This provides a potential mechanism explaining the cell cycle arrest and differentiation inhibition observed in this study upon *ALDOA* overexpression: *ALDOA* may directly interfere with the transcriptional regulation of key cell cycle genes and myogenic differentiation genes through its nuclear functions.

This study found that overexpression of *ALDOA* in two cell types inhibited their proliferation. CCK-8 and EdU assays demonstrated that *ALDOA* overexpression significantly reduced cell viability and proliferation rates. Flow cytometry further revealed that it induced G0/G1 phase arrest and inhibited S phase entry. This result contradicts reports of *ALDOA* promoting proliferation in certain tumor cell lines, leading us to infer that the function of this gene may exhibit cell type and context specificity [13,17]. In rapidly proliferating cancer cells, the high expression of *ALDOA* better meets their substantial biosynthetic and energy demands [18]. However, in normal myoblasts, their proliferative capacity is limited, and they ultimately need to exit the cell cycle to initiate cellular differentiation. We hypothesize that *ALDOA* overexpression may mimic a “metabolic checkpoint” signal, forcing premature exit from the cell cycle. Studies indicate that metabolic enzyme expression can activate signaling pathways such as p53 or AMPK, thereby inducing cell cycle arrest [19,20]. Our study revealed a seemingly paradoxical phenomenon: while endogenous *ALDOA* expression increases during normal differentiation, its premature overexpression strongly inhibits the process. This underscores that the timing and level of *ALDOA* expression must be precisely regulated for successful myogenesis. We propose a ‘metabolic gatekeeper’ hypothesis to reconcile these findings: during normal differentiation, the gradual upregulation of *ALDOA* supports the necessary glycolytic flux for myotube maturation. However, premature and supraphysiological overexpression, as in our gain-of-function model, may disrupt the metabolic dynamics and mimic a ‘differentiation-incompatible’ metabolic state, potentially through aberrant nuclear signaling. This dysregulation could prematurely activate cell cycle arrest pathways or directly interfere with the transcriptional activity of myogenic master regulators like *MYOD* and *MYOG*, thereby halting the differentiation program. This hypothesis aligns with models suggesting that a specific window of glycolytic activity is crucial for coordinating cell fate decisions [20,21]. Simultaneously, we observed that overexpression of the *ALDOA* gene leads to downregulation of proliferation markers such as *CDK2*, *Cyclin D1*, and *KI67*. *CDK2* [22] and *Cyclin D1* are key regulators of the G1/S transition; their downregulation directly causes cell cycle arrest. Furthermore, the localization of *ALDOA* within the cell nucleus may enable its involvement in regulating the transcription of cell cycle-related genes, such as by interacting with E2F or p53 to influence the expression of their target genes [23]. Therefore, *ALDOA* may upregulate cell cycle inhibitors or downregulate cyclin through an as-yet-unidentified signaling pathway in myoblasts, leading to G1/S phase arrest. This will be a key focus for future research.

Research has revealed that overexpression of *ALDOA* significantly promotes apoptosis in C2C12 and porcine skeletal muscle cells, demonstrating its dual role in regulating cellular fate. Although *ALDOA* primarily functions as a glycolytic enzyme, recent studies have shown it also plays a crucial role in regulating oxidative stress and mitochondrial function [14,24]. Overexpression of *ALDOA* may disrupt normal metabolic homeostasis, leading to accumulation of intermediate metabolites or abnormal ATP/ADP ratios. This triggers mitochondrial dysfunction and ultimately initiates the caspase cascade, resulting in apoptosis [23]. Previous studies have indicated that the *ALDOA* gene may participate in and support various muscle-related biological processes and cellular functions [25]. Its abnormal expression may disrupt the cytoskeleton, thereby inducing a form of cell death known as “apoptosis by loss of anchorage [26].” In muscle cells attempting to differentiate, this disruption of the cytoskeleton is particularly detrimental.

Our experimental results indicate that endogenous *ALDOA* expression increases with differentiation, consistent with the requirement for muscle cells to shift from oxidative phosphorylation to glycolytic metabolic pathways to support their contractile function [8]. However, gain-of-function experiments indicate that *ALDOA* expression levels require precise regulation. Its premature overexpression strongly inhibits the transcriptional activity of key myogenic regulators (*MYOD*, *MYOG*, *MEF2C*), thereby impairing myotube formation. *MYOD* and *MYOG* serve as primary regulators of myogenic differentiation, initiating and coordinating the entire myogenesis program [27,28]. As a speculative mechanism, nuclear *ALDOA* could potentially influence the epigenetic landscape, for instance by modulating metabolite pools like α-KG that affect TET enzyme activity and thereby DNA methylation at loci such as the *MYOD* promoter [29]. However, this remains highly hypothetical, and future work must prioritize testing whether ALDOA’s primary nuclear role involves direct DNA binding or indirect modulation of transcription factors.

Through this study, we discovered that *ALDOA* plays a novel functional role in skeletal muscle cells, and its expression levels must be strictly regulated to ensure normal myogenesis. Dysregulation of its function disrupts muscle cell homeostasis by interfering with cell cycle progression, inducing apoptosis, and suppressing myogenic transcription. Future investigations could focus on elucidating the molecular mechanisms by which *ALDOA* inhibits *MYOD/MYOG* expression—particularly whether this involves nuclear translocation and direct transcriptional regulation—to explore its impact on muscle differentiation in greater detail. Concurrently, examining the upstream signaling pathways that trigger cell cycle arrest and apoptosis could provide alternative avenues for exploring its potential therapeutic applications. We currently observe the potential steering effect of *ALDOA* overexpression on cell fate. To definitively confirm *ALDOA*’s essential physiological function and rule out potential artifacts from overexpression, future studies must incorporate loss-of-function experiments, such as knocking down or knocking out *ALDOA* using siRNA, shRNA, or CRISPR/Cas9 technology. Should *ALDOA* knockdown promote myocyte proliferation and differentiation, this would corroborate our findings, establishing a comprehensive evidence chain to confirm *ALDOA*’s intrinsic negative regulatory role in muscle development.

## 5. Conclusions

The *ALDOA* gene exhibits high expression and strong conservation in muscle tissues. Functional experiments demonstrate that *ALDOA* overexpression significantly inhibits proliferation in C2C12 and porcine skeletal muscle satellite cells, causing cell cycle arrest and downregulating proliferation-related genes such as *CDK2* and *Cyclin D1*. Collectively, gain-of-function studies demonstrate that *ALDOA* overexpression negatively regulates skeletal muscle cell growth and development by suppressing proliferation, promoting apoptosis, and impeding differentiation in both C2C12 and primary porcine satellite cells, albeit with some cell type-dependent nuances, highlighting the context-dependent functionality of *ALDOA*.

## Figures and Tables

**Table 1:** The primer sequences of PCR.

Primer	Sequence
*ALDOA*-Forward (5′-3′)	ATGGCAAAGCGCGAGCCG
*ALDOA*-Reverse (5′-3′)	TTAGTAGGCATGGTTAGAGATGAAG

**Table 2:** The primer sequences of RT-qPCR.

Gene	Forward (5′-3′)	Reverse (5′-3′)
Mus-*ALDOA*	CGGCTGTCTACAAGGCTCTGAG	CTGTGCGACGAAGTGCTGTG
Mus-*MYOD*	TCCAACTGCTCTGATGGCATGATG	ACTGTAGTAGGCGGTGTCGTAGC
ciMus-*MYOG*	GAGACATCCCCCTATTTCTACCA	GCTCAGTCCGCTCATAGCC
Mus-*MEF2C*	AAGCCTCAGCATCAAGTCAGAACC	GCGTGGTGTGTTGTGGGTATCTC
Mus-*β-actin*	GTGACGTTGACATCCGTAAAGA	GCCGGACTCATCGTACTCC
Sus-*ALDOA*	CCATCAACCTCAACGCCATCAAC	AGGGCTCGCTTGACATATTCTTCC
Sus-*MYOD*	CCGCTCCGCGACGTAGATT	GGCAGGGAAGTGCGAGTGTT
Sus-*MYOG*	AAACTACCTGCCCGTCCACCTC	GGTCCCCAGCCCCTTATCTTCC
Sus-*MEF2C*	TTGGGTTGATGAAGAAGGCTTAGAG	GCTTGTTGGTGCTGTTGAAGATG
Sus-*β-actin*	GATGACGATATTGCTGCGCTCG	AGCTCGTTGTAGAAGGTGTGG

## Data Availability

All the original data involved in this experiment can be obtained from the author. For details, please contact e-mail hongzhencao@163.com.

## Supplementary Materials

The following supporting information can be downloaded at: https://www.mdpi.com/article/10.3390/genes16101186/s1, Figure S1: Comparison of amplified sequencing results with NCBI entries; Figure S2: Conserved sequence alignment of the ALDOA gene; The “*” in Figure C indicates the conserved sequence of this gene; Figure S3: Localization of the ALDOA gene in C2C12 cells at different time points; Figure S4: Localization of the ALDOA gene in porcine skeletal muscle satellite cells at different time points; Figure S5: EdU assay for cell proliferation-C2C12; Figure S6: EdU assay for cell proliferation- Skeletal Muscle Satellite Cells.
