# Supplementary material for: Functional Validation of ALDOA in Regulating Muscle Cell Fate: Based on In Vitro Proliferation, Apoptosis, and Differentiation Experiments
Source: Genes (Basel). 2025 Oct 12;16(10):1186. doi: 10.3390/genes16101186 (PMC12563186; doi:10.3390/genes16101186)
Supplement: Supplementary file 1 [file genes-16-01186-s001.zip › genes-3925144-SM.pdf]

Supplementary Materials:

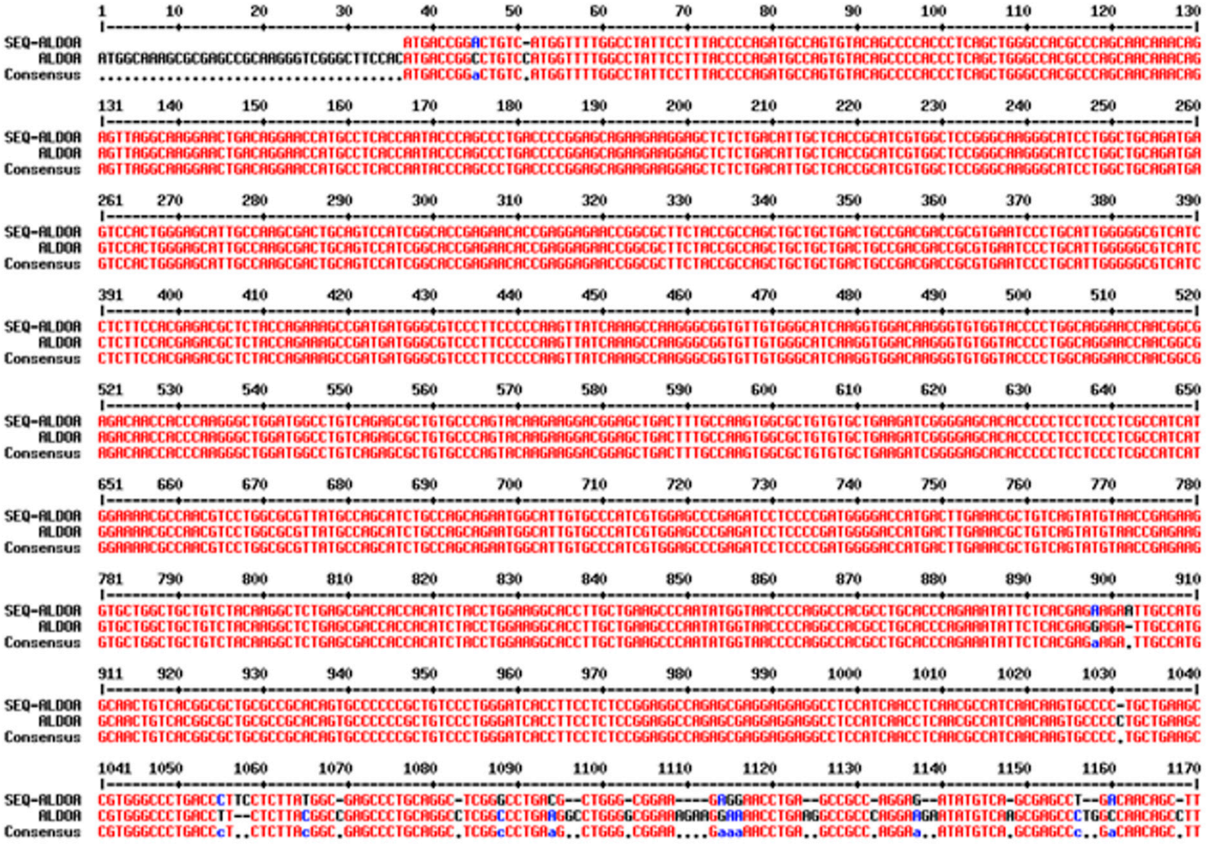

Figure S1. Comparison of amplified sequencing results with NCBI entries.

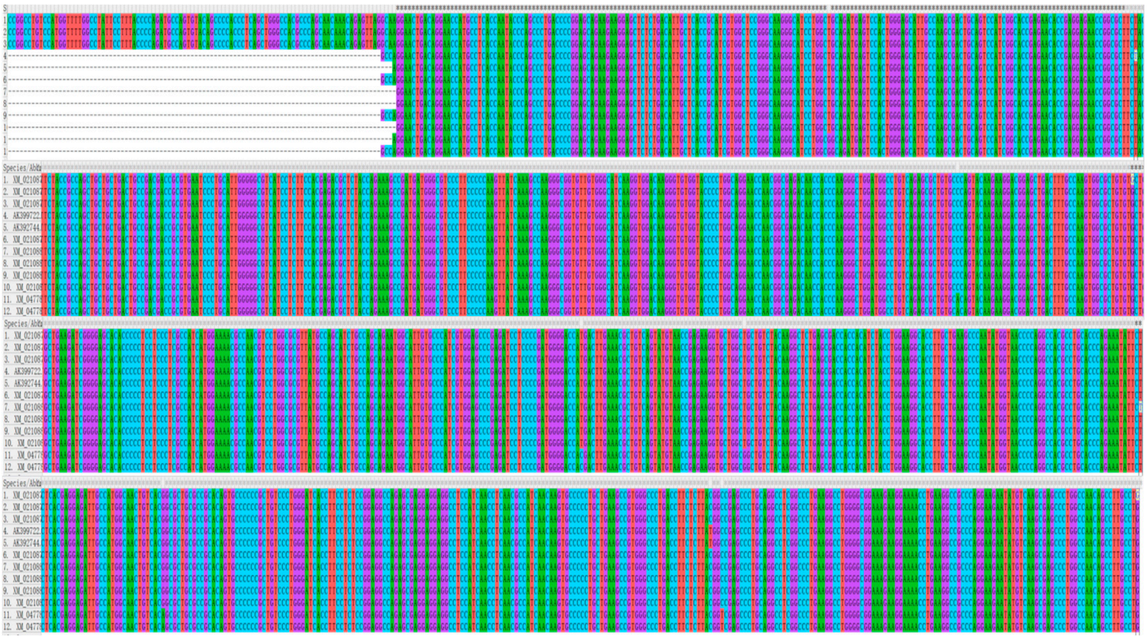

Figure S2. Conserved sequence alignment of the *ALDOA* gene; The "*" in Figure C indicates the conserved sequence of this gene.

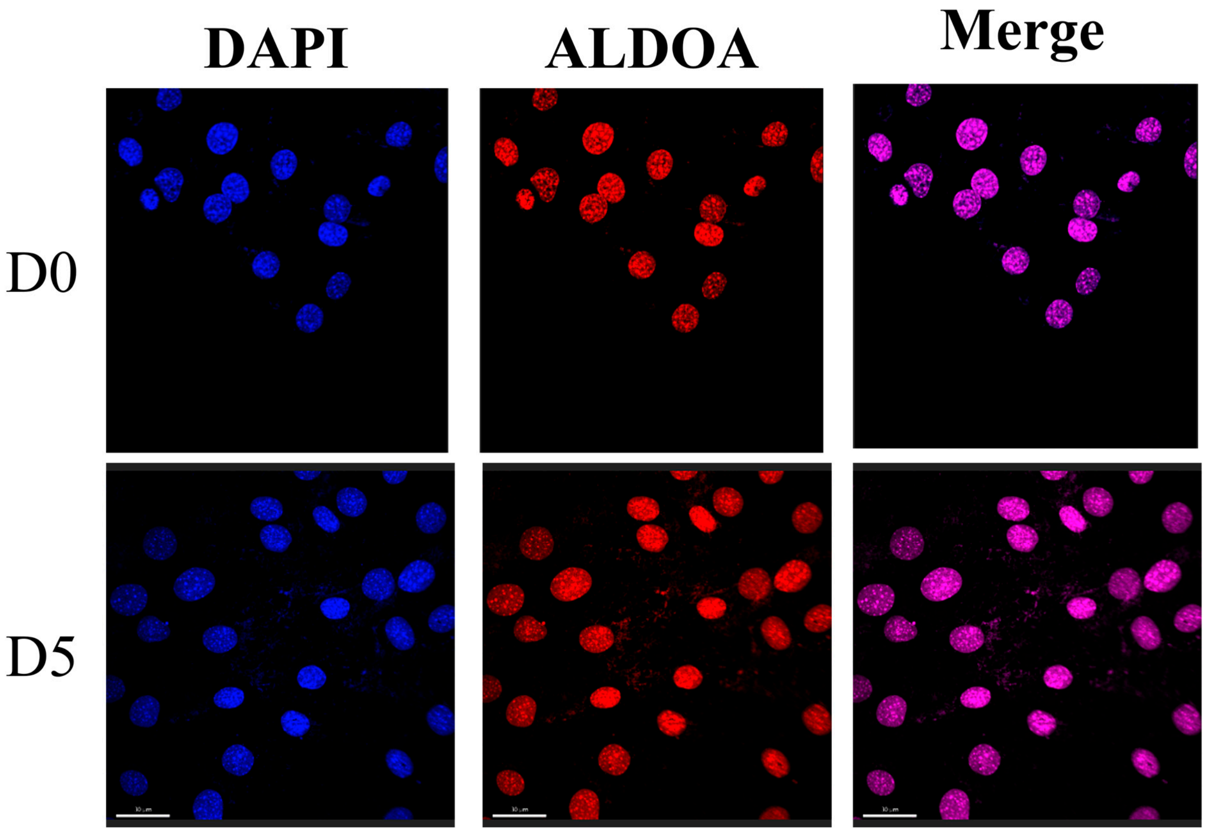


Figure S3. Localization of the *ALDOA* gene in C2C12 cells at different time points.

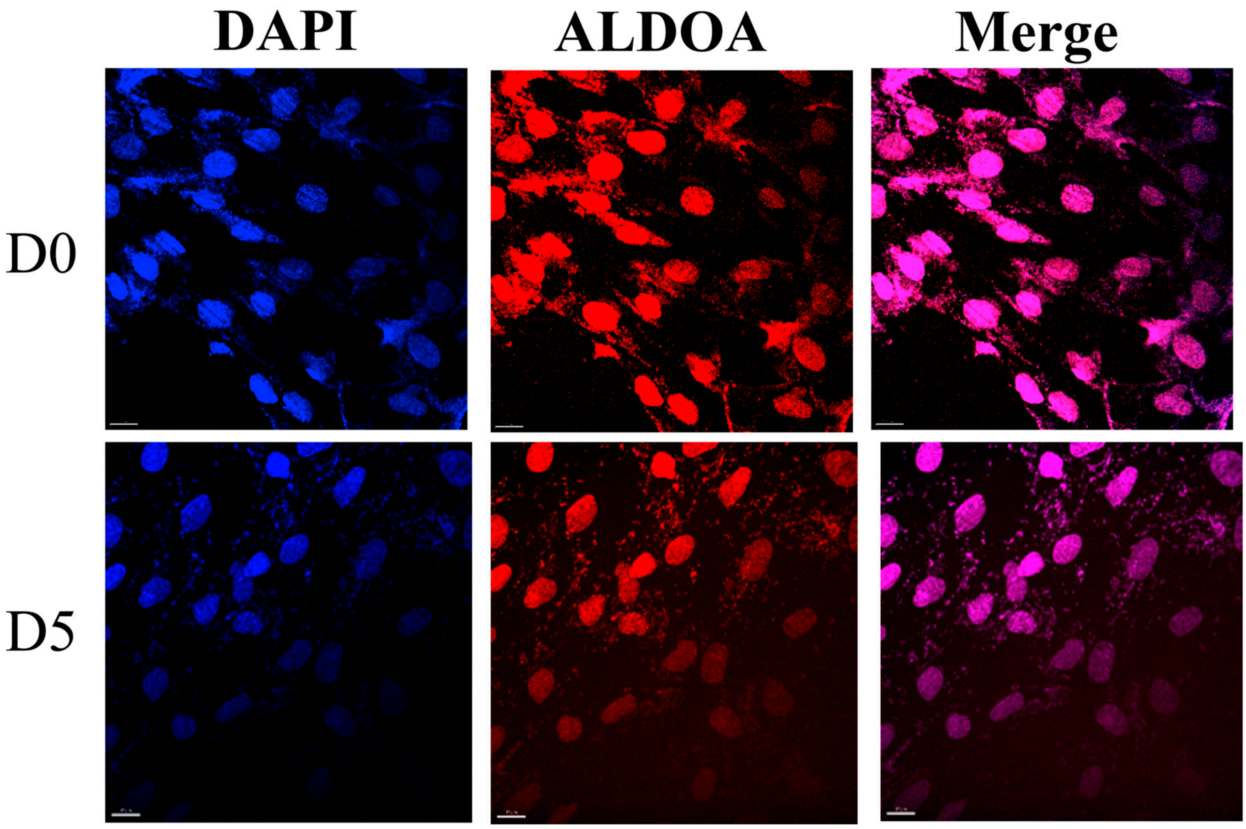


Figure S4. Localization of the *ALDOA* gene in porcine skeletal muscle satellite cells at different time points.

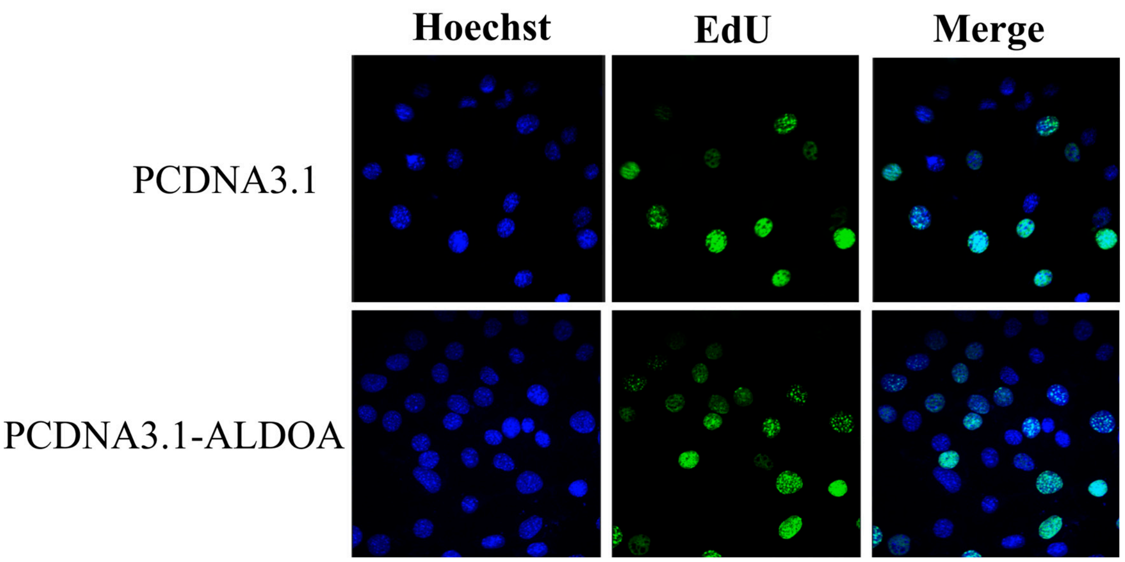


Figure S5. EdU assay for cell proliferation-C2C12.

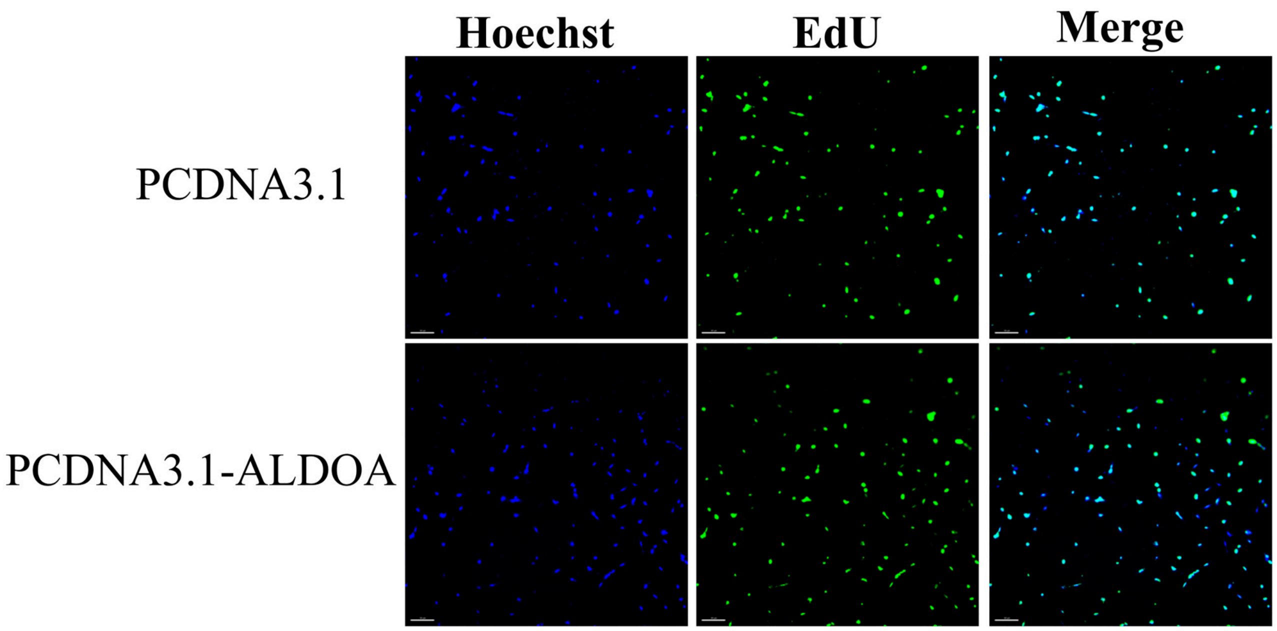


Figure S6. EdU assay for cell proliferation- Skeletal Muscle Satellite Cells.
